# Validation of the short Arabic UPPS-P Impulsive Behavior Scale

**DOI:** 10.1186/s12888-017-1407-y

**Published:** 2017-07-06

**Authors:** Ghada Bteich, Djamal Berbiche, Yasser Khazaal

**Affiliations:** 1 Lebanese University Faculty of Public Health, Lebanon University, Tripoli, Lebanon; 2 Charles-LeMoyne Hospital Research Centre, Sherbrooke University, Sherbrooke, Canada; 3 Geneva University Hospitals, Grand-Pré 70C, 1206 Geneva, Switzerland; 4 University of Geneva, Geneva, Switzerland; 5 University Institute of Mental Health at Montreal, Montreal, Canada

**Keywords:** Impulsivity, UPPS, CIUS, Internet addiction, Validation

## Abstract

**Background:**

Impulsivity is involved in numerous psychiatric and addictive disorders, as well as in risky behaviors. The UPPS-P scale highlights five complementary impulsivity constructs (i.e., positive urgency, negative urgency, lack of perseverance, lack of premeditation, and sensation seeking) that possibly work as different pathways linking impulsivity to other disorders. In this study, we aimed to evaluate the psychometric properties of the Arab language short 20-item UPPS-P scale and to eventually validate it.

**Methods:**

Participants were recruited online through e-mail invitations. After online informed consent was obtained, the questionnaires (the UPPS-P and the Compulsive Internet Use Scale [CIUS]) were completed anonymously. The five dimensions of the Arab UPPS-P model were assessed in a sample of 743 participants.

**Results:**

As in other linguistic assessments of the UPPS-P, confirmatory factor analysis showed the validity of a model with five different, but nonetheless interrelated, facets of impulsivity. A three-factor model with two higher order factors—urgency (negative and positive) and lack of conscientiousness (lack of premeditation and lack of perseverance)—and a third sensation seeking factor fit the data well, but to a lesser extent. The results suggested good internal consistency, with external validity shown from correlations between some of the UPPS-P components and a measure of addictive Internet use (the CIUS).

**Conclusion:**

The Arab short UPPS-P is a valid assessment tool with good psychometric properties and is suitable for online use.

## Background

Impulsivity is involved, for different aspects, in a wide range of psychiatric disorders and behavioral disturbances [1]), such as self-harm [2], substance use disorders [3, 4], behavioral addictions [5, 6], bulimia [2], borderline personality disorder [7], and attention-deficit/hyperactivity disorder [8, 9]. The concept of impulsivity is, however, an umbrella construct that comprises a combination of various dimensions [10–12].

Different authors and models have tried to capture the many components of impulsivity in several questionnaires according to various conceptions [13]. In an attempt to overcome the weakness linked to this range of questionnaires and, to some extent, the inconsistencies among the conceptualizations of impulsivity, Whiteside and Lyman [14] conducted a factor analysis on the main questionnaires assessing impulsivity. From this work [14] and from a number of successive works [15, 16] emerged the 59-item UPPS-P Impulsive Behavior Scale with five different facets of impulsivity:

1. Negative urgency (to act impulsively while facing negative emotional situations).
2. Positive urgency (to act impulsively while facing intense positive emotional situations).
3. Lack of premeditation (the propensity to not take into account the results of an act before engaging in that act).
4. Lack of perseverance (difficulty in staying focused on hard or boring tasks).
5. Sensation seeking (a tendency to pursue exciting activities and an openness to risky and unconventional activities).

Numerous studies showed that the UPPS-P facets of impulsivity were associated, in different ways, with several psychiatric disorders: addiction, problematic behaviors, and self-harm [17–20]. For instance, urgency seems to be particularly involved in addictive behaviors [4]. The UPPS-P appears, however, to function comparably across genders [21]. These findings highlight the importance of distinguishing between different aspects of impulsivity.

A short 20-item version of the UPPS-P was recently developed [22] and validated in different languages (i.e., French, Spanish, English, Italian; [22–24]. These studies showed that the psychometric properties of the short version of the UPPS-P have a strong factorial structure similar to that of the original scale. Two different models were reported to fit the data well, the most frequently found model having five distinct but related impulsivity facets, as described earlier [22–25]. In addition, some studies found adequate fit for a hierarchical model with two higher order factors of urgency (resulting from positive and negative urgency) and lack of conscientiousness (resulting from lack of premeditation and lack of perseverance; [22, 24, 25].

This second model is congruent with the results of a recent meta-analysis [12] on the psychopathological correlates of the UPPS-P facets that found that negative urgency offers the greatest correlational effect sizes across all studies.

A similar pattern of correlations was found with positive urgency, showing similarities to those displayed with negative urgency. Comparable correlational patterns were obtained for the lack of perseverance and lack of premeditation pathways. The results of this meta-analysis converge with the possibility that the two higher order factors are important in addition to the five-factor model.

Despite the increasing importance of the 20-item version of the UPPS-P scale, it has not yet been studied in the Arab language. The aim of the present study was to develop a 20-item short Arab UPPS-P (S-UPPS-P) and to explore its psychometric properties, including its factorial structure, internal consistency, and external validity.

## Methods

### Participants and procedure

The sample comprised 743 participants (73.4% female). Participants were students and collaborators from the Lebanese University recruited online through e-mail invitations. The local institutional review board of the Lebanese University approved the study. An e-mail was sent out to all students and collaborators through the university e-mail system. Before the participants completed the survey, a form was provided explaining the purpose of the study and assuring them that data collection, storage, and reporting techniques would protect confidentiality and anonymity. Participants gave online informed consent and the questionnaires were completed anonymously. No compensation was given.

The age (mean) distribution of the participants was as follows: 18–25 years (63.68), 26–30 (13.75), 31–40 (13.31), 41–49 (5.64), 50–60 (3.18), and >60 (0.43). Participants completed an online survey in Arab that included age, gender, the S-UPPS-P, and the Arab version of the Compulsive Internet Use Scale (CIUS) [26]. The number of data points available from the questionnaire subscales used to establish construct validity was variable (Table 1). The mean, standard deviation, internal consistency (Cronbach’s alphas), and correlations between subscales are reported for all questionnaires in Table 1.

**Table 1:** Descriptive statistics, internal consistency (Cronbach’s α), and Pearson correlations among the subscales of the Arabic S-UPPS-P and the CIUS

Questionnaire	*N*	Mean	*SD*	α	1	2	3	4	5	6
1. UPPS — negative urgency	658	9.74	2.22	.63	1.00					
2. UPPS — positive urgency	661	10.67	2.10	.63	0.55^a^	1.00				
3. UPPS — lack of premeditation	700	7.35	2.23	.58	0.29^a^	0.22^a^	1.00			
4. UPPS — lack of perseverance	656	7.60	1.87	.72	0.15^a^	.07^b^	0.41^a^	1.00		
5. UPPS — sensation seeking	660	9.56	2.51	.70	0.17^a^	.24^a^	0.17^a^	0.12^c^	1.00	
6. CIUS	614	20.66	9.16	.81	0.29^a^	0.25^a^	0.14^d^	0.18^a^	0.19	1.00

*Note. UPPS* UPPS Impulsive Behavior Scale, *CIUS* Compulsive Internet Use Scale

^a^ *p* < .0001

^b^ *p* = .09

^c^ *p* = .002

^d^ *p* = .0004

### Measures

#### The Arab S-UPPS-P impulsivity scale

This scale is a 20-item questionnaire that evaluates five facets of impulsivity: positive urgency, negative urgency, lack of perseverance, lack of premeditation, and sensation seeking. Four items on a 4-point Likert scale evaluate each of the five facets. To develop the Arabic S-UPPS-P, we had the 20 items of the original S-UPPS-P translated by a professional translator from French into Arabic, and then back-translated (by the authors GB and YK) into Arabic. All differences identified between the original S-UPPS-P and the back-translation were examined until an adequate resolution was reached.

#### The Arab CIUS [26]

This scale is the Arabic validation of the CIUS [27]; it is a questionnaire that aims to assess on a continuum (using Likert scales) the severity of the supposed main symptoms of problematic excessive Internet use (i.e., loss of control, preoccupation, withdrawal, salience, conflict, and coping). Like the original version [27], the Arab CIUS is a 14-item questionnaire with a good unidimensional factorial structure [26]. The Arab CIUS was chosen to add external validity to the S-UPPS-P validation study. The scale was used here because of the wide use of the Internet in Lebanon and Arab-speaking countries [28, 29], the availability of the scale in Arabic, its shortness (useful for online studies), and the links previously shown with some measures of addictive Internet use and the S-UPPS-P [5, 6, 30].

### Statistical analyses

To assess the factor structure of the Arabic S-UPPS-P, we used SAS 9.3 (SAS Institute, Inc., Cary, NC, USA) to perform a confirmatory factor analysis (CFA) and LISREL 8.80 for Windows to analyze the covariance matrix. We ran three models: a single unitary impulsivity construct, a model with five interrelated constructs, and a model that involves three interrelated constructs (urgency: negative and positive, conscientiousness: lack of premeditation and lack of perseverance, and sensation seeking).

We assessed goodness of fit by using the χ^2^ statistic, where acceptable fit is indicated by a nonsignificant value. This statistic spreads, however, with sample size; furthermore, it is rarely nonsignificant when CFAs are carried out on self-administered questionnaires [31]. Therefore, we reported the following additional indices: the ratio of χ^2^ to degrees of freedom (*df*), the root mean square error of approximation (RMSEA), the comparative fit index (CFI), the adjusted goodness-of-fit index (AGFI), the normed fit index (NFI), and the non-normed fit index (NNFI). A χ^2^/*df* < 5, RMSEA < .08, CFI > .95, AGFI > .85, NFI > .90, and NNFI > .95 are considered excellent fit. In addition, we computed the expected cross-validation index (ECVI) in order to compare the three models. The ECVI evaluates whether a given model has similar validation in different samples of the same population of the same size. A small ECVI indicates good likelihood of replication.

Two-tailed Pearson correlations were furthermore performed to assess the links between the five UPPS-P dimensions and the Arab CIUS.

## Results

We found that the one-factor model had a poor fit (χ^2^ (169) = 1658.95, *p* < .001, χ^2^/*df* = 9.82, RMSEA = .1166, CFI = .453, AGFI = .681, NFI = .4303, NNFI = .3846), whereas the five-factor model (intercorrelated) had an excellent fit (χ^2^ (155) = 439.4, *p* < .001, χ^2^/*df* = 2.84, RMSEA = .0532, CFI = .8955, AGFI = .9146, NFI = .9491, NNFI = .8719) and the three-factor model (intercorrelated) had an adequate fit (χ^2^ (164) = 601.7, *p* < .001, χ^2^/*df* = 3.67, RMSEA = .0642, CFI = .8392, AGFI = .8855, NFI = .7933, NNFI = .8137).

According to the ECVI statistics, the five-factor model is more adequate (ECVI = 0.8536) than the three-factor model (ECVI = 1.0754). The one-factor model is least adequate (ECVI = 2.6909).

**Fig. 1:**
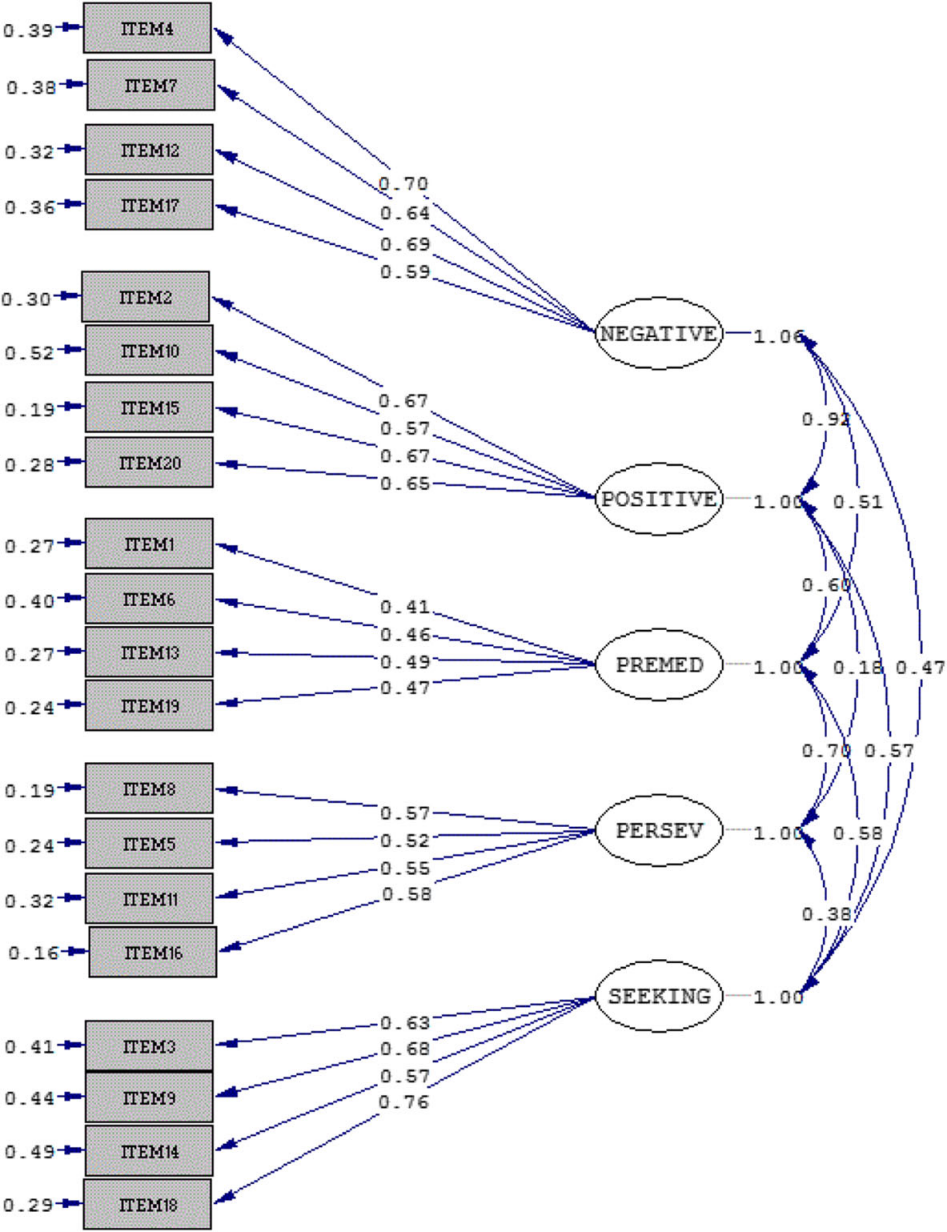
The five-factor model is presented. Error variance and factor loadings are shown with one-way arrows. Correlations between variables (taking into account covariance between items) are shown via two-way arrows. NEGATIVE = negative urgency; POSITIVE = positive urgency; PREMED = lack of premeditation; PERSEV = lack of perseverance; SEEKING = sensation seeking

The retained model, item loadings, and intercorrelations are reported in Fig. 1. The number of participants (N), means, and standard deviations are reported for the five UPPS-P facets and for the CIUS in Table 1. As shown, Cronbach’s α ranged from .58 to .81, indicating good internal consistency, as found in previous assessments of the characteristics of the S-UPPS-P [22, 25]. Between-variable correlations are also specified in Table 1.

## Discussion

This study assessed the psychometric characteristics of the Arab S-UPPS-P, used online, in a sample of university students and collaborators. To the best of our knowledge, this is the first study on the validation of the S-UPPS-P in Arabic. Furthermore, it is one of the largest studies on the psychometric properties of the S-UPPS-P.

The psychometric properties of the S-UPPS-P have been repeatedly assessed in different linguistic versions. Thus, from the results of previous studies, we chose to use CFA analyses rather than exploratory analyses because of the availability of a priori hypotheses.

The main findings can be summarized as follows. First, the Arab S-UPPS-P showed the same theory-driven factor structures reported in previous S-UPPS-P validation studies [22–25], composed of five interrelated dimensions. Second, the internal consistency of the subscales is good (Cronbach’s α ranges from .58 to .81) and has a similar range to that in the other studies except for the lack of premeditation subscale (slightly lower Cronbach’s α). Third, construct validity was confirmed by the specific correlations shown with the CIUS score (Table 1). Statistically significant correlations were observed between the CIUS scale and each of the S-UPPS-P subscales. Furthermore, differences regarding the importance of the correlations were observed from .18 to .29, arguing for possible overlapping but distinct impulsivity pathways measured by the S-UPPS-P. The strongest associations were observed between CIUS and the urgency subscales, as found in other studies reporting the links between S-UPPS-P and addictive disorders [4, 5]. In contrast, a previous study on the links between the CIUS and the Italian S-UPPS-P reported a significant correlation (.031) with only the positive urgency subscale between the S-UPPS-P subscales and the CIUS. The correlation with the negative urgency subscale failed to reach significance, which was thought to be due to the small sample size.

Such results are congruent with those of other studies on the links between the UPPS model and addictive behaviors. For instance, a recent meta-analysis [32] on the relationship between the UPPS model and alcohol use showed differential effects of the different subscales. Drinking problems were most highly related to negative and positive urgency. In a study on Internet gambling, [33] also found that the different dimensions of the UPPS-P were involved in the most problematic gambling, except for the sensation seeking dimension.

The results of our study, similar to those of other studies related to the validation of the S-UPPS-P [22, 24, 25], show that a three-factor model—urgency (negative and positive), lack of conscientiousness (lack of premeditation and lack of perseverance), and sensation seeking—fits the data well, but to a lesser extent than the five-factor model. This result is in concordance with the findings of a number of studies and meta-analyses [12].

The main limitation of the present study is that the sample was composed mainly of university students, which limits its representativeness. Several other limitations reduce the generalizability to the Arab population of the results. The sample included a majority of female (73,4%) and was recruited online leading to possible recruitment biases [34]. Furthermore, details about the exact level of instruction were not collected.

In consideration of the associations found between the CIUS score and the different S-UPPS-P facets, further studies may increase the understanding of such associations by using a more detailed assessment of Internet-related activities (e.g., social network, online gaming, cyberporn) and the related motives for their use (e.g., coping, social). A wider assessment of possible comorbid mental illness and prospective studies are also needed to better understand such associations.

## Conclusions

Overall, the results of the present study suggest that the Arab S-UPPS-P has adequate psychometric properties that are similar to those found in studies on other linguistic validations of the scale [22–25]. Our study thus showed that the Arab S-UPPS-P is a useful and valid short questionnaire for assessing impulsivity components in clinical practice and research.

## Abbreviations

AGFI: Adjusted goodness-of-fit index
CFI: Comparative fit index
CIUS: Compulsive Internet Use Scale
*df*: Degrees of freedom
ECVI: Expected cross-validation index
NFI: Normed fit index
NNFI: Non-normed fit index
RMSEA: Root mean square error of approximation
S-UPPS-P: Short version (20 items) of the UPPS-P
UPPS-P: Acronym for the Impulsive Behavior Scale with five 5 different facets of impulsivity: negative urgency, positive urgency, lack of premeditation, lack of perseverance and sensation seeking
